# Association between body mass index, multi-morbidity and activities of daily living among New Zealand nursing home older adults: a retrospective analysis of nationwide InterRAI data

**DOI:** 10.1186/s12877-021-02696-8

**Published:** 2022-01-19

**Authors:** Isaac Amankwaa, Katherine Nelson, Helen Rook, Caz Hales

**Affiliations:** 1 Centre for Health and Social Practice, Waikato Institute of Technology, Hamilton, New Zealand; 2 Faculty of Health, School of Nursing, Midwifery, and Health Practice, Victoria University of Wellington, Wellington, New Zealand

**Keywords:** Activities of daily living, Aged care, Body mass index, Multi-morbidity, Nursing home, Obesity, Older adults, Residents

## Abstract

**Background:**

Obesity is a well-established risk factor for multi-morbidity and disability among older adults in the community and acute care settings. However, nursing home residents with body mass index (BMI) below 18.5 kg/m^2^ and above 25.0 kg/m^2^ have been understudied. We examined the prevalence of multi-morbidity and disability in activities of daily living (ADL) by BMI category and further investigated the association between BMI, multi-morbidity, and disability of ADL in a large cohort of older adults in nursing homes in New Zealand.

**Methods:**

A retrospective review of nursing home residents’ data obtained from the New Zealand International Resident Assessment Instrument national dataset from 2015 to 2018. One hundred ninety-eight thousand seven hundred ninety older adults (≥60 years) living in nursing homes were included. BMI was calculated as weight in kilograms (kg) divided by height in meters squared (m^2^). Multimorbidity was defined as the presence of ≥2 health conditions. The risk of disability was measured by a 4-item ADL self-performance scale. The prevalence ratio (PR) of the association between BMI and multi-morbidity and between BMI and disability in ADL was assessed using Poisson regression with robust variance.

**Results:**

Of the 198,790 residents, 10.6, 26.6, 11.3 and 5.4% were underweight, overweight, obese, and extremely obese, respectively. 26.4, 31.3 and 21.3% had one, two and three disease conditions, respectively, while 14.3% had four or more conditions. 24.1% could perform only one ADL, and 16.1% could perform none. The prevalence of multi-morbidity increased with increasing BMI, whereas mean disability in ADL decreased with increasing BMI. The risk of multi-morbidity was higher for the overweight (PR, 95%CI: 1.03, 1.02–1.03) and obese (PR, 95% CI: 1.07, 1.06–1.08) compared to normal weight after controlling for age, sex, ethnicity, and region. BMI was inversely associated with mean ADL; β, 95% CI for overweight (− 0.30, − 0.32, − 0.28) and obese − 0.43, − 0.45, − 0.40 compared to normal weight.

**Conclusion:**

Being underweight was associated with a decline in the performance of ADL in nursing home residents. In contrast, being overweight and obese positively affected functional performance, demonstrating that the obesity paradox plays an important role in this population. The observed associations highlight areas where detection and management of underweight and healthy aging initiatives may be merited.

## Introduction

There is an increasing prevalence of obesity amongst older adults globally and within New Zealand; obesity rates in 65–74-year-olds and 75 years and older are estimated to be 38.3 and 26.5%, respectively [1]. Studies conducted among older adults, mainly in acute hospital and community settings, suggest that an increase in body mass index (BMI) and being older (≥65 years) predisposed an individual to multi-morbidity [2, 3], cardiometabolic comorbidity [4, 5] and decubitus ulcers [6]. Other studies have reported that overweight and class I obese (BMI 30–34.9 kg/m^2^) among older adults have some protection against comorbidity [7, 8] and myocardial infarction [9]. Despite the prevailing inconsistencies, research involving the aged population has predominantly focused on the effect of BMI on mortality. It has paid limited attention to other measures of health, such as the effect of BMI on disability [10] and activities of daily living (ADLs).

The effect of BMI categories on disability in older populations is poorly understood. Longitudinal [10–14] and cross-sectional [15–18] studies involving community-dwelling adults (≥65 years) found obesity to be an independent risk factor for functional disability. In contrast, studies focusing on older adults (≥ 60 years) in nursing home residents [19–21] and one study of community-based older adults aged ≥80 years [22] reported that a higher BMI significantly decreases the risk of disability in ADL. The controversy in findings may be linked to age, setting, health and nutritional status [18, 23]. Kaiser et al. [20] report that the controversial relationship between normal and low BMI ranges and functional status might apply specifically to nursing home residents and not independently living older adults.

Previous research has been limited to the acute hospital setting and community-dwelling older adults, mainly in North America [10, 24], Europe [11, 14], and Asia [12, 13, 22]. As a result, there remains a paucity of evidence on the prevalence of BMI categories and their association with ADL disabilities and multi-morbidity in nursing homes in general. Furthermore, the few studies that have examined these relationships in nursing homes have been limited by small sample sizes (< 300), estimating BMI by self-report, and captured only a subset of BMI categories (i.e., BMI ≥ 28 kg/m^2^). We designed a study to address these limitations. Our study analysed data across the entire spectrum of BMI, from underweight to extreme obesity. It utilised objectively recorded InterRAI data from rest home residents aged 60 years and above in New Zealand. The study’s objective was to determine the prevalence of multi-morbidity and disability of ADL by BMI category and assess the association between BMI, multi-morbidity, and disability of ADL in older adults in nursing homes in New Zealand. As obesity, multi-morbidity and ADLs depend not only on BMI but also on age, sex, and ethnicity; we controlled for these factors.

## Methods

### Participants

For this retrospective study, resident data were obtained from the New Zealand International Resident Assessment Instrument (InterRAI) national dataset from 2015 to 2018. This data reports on care provided to all residents in nursing homes in New Zealand who consented to have their anonymised data available for research purposes; consent rate for our study dataset was 81.5%. All residents are required to have a long-term care facility assessment completed every 6 months, or when a significant change in health has occurred as part of a mandated nationally standardised assessment [25]. In total, 224,200 residents aged 60 years and over were included in the dataset.

### Data collection

#### The interRAI-LTCF assessment tool

The datasets used for defining outcome measures are based on the New Zealand version of the interRAI-Long Term Care Facility tool [26]. This tool includes the following outcome measures: Activities of Daily Living (ADL) scales, ADL self-performance hierarchy, Aggressive Behaviour Scales, Cognitive Performance Scale, Depression Rating Scales, Body Mass Index (BMI), Communication Scale, Pain Scale and Changes in Health, End-Stage Disease, Sign and Symptoms (CHESS score) [27]. Together, the interRAI tool contains more than 350 variables that include social, demographic, medical (e.g., disease diagnosis), care programs and medications. The dependent variables used to inform this study’s analysis are scores of ADL, BMI, and disease diagnosis. The interRAI assessments are completed by health professionals for whom assessment forms part of their scope of practice, hold a current annual practicing certificate, have completed the InterRAI assessor training course, and are endorsed as competent for the relevant assessment instrument [28]. Are routinely re-tested to ensure that high-quality assessment is maintained [25]. The above measures ensure that the interRAI-LTCF records represent a robust data set regarding the older adults in New Zealand.

##### Definitions and outcome measures

Disability was assessed using an internationally validated instrument that measures ability in ADL to assess physical function. The 4-item ADL self-performance scale measures personal hygiene, locomotion/movement, toilet use, and eating. A potential difficulty for each of the four items was scored from 0 (independence or no functional difficulty) to 4 (total dependence). The scale categorizes ADL according to the stage of disability that a person had reached: supervision required, limited assistance, extensive assistance required, maximal assistance required, dependent and totally dependent [29, 30]. We defined disability in ADL as participants identified as dependent in all ADL categories (personal hygiene, locomotion/movement, toilet use, and eating). We created a binary dependent variable for the Poisson regression analysis (1 = Disability in ADL; 2 = Not disabled in ADL).

Multimorbidity was defined as having two or more chronic diseases. Body mass index (BMI), as defined by World Health Organization [31], was calculated as weight in kilograms (kg) divided by height in meters squared (m^2^). BMI was further categorized into underweight (< 18.5 kg/m^2^), normal weight (18.5 to 24.9 kg/m^2^), overweight (25.0 to 29.9 kg/m^2^), obese (30.0 to 39.9 kg/m^2^) and extremely obese (≥40.0 g/m^2^) [31].

### Statistical analysis

The InterRAI aggregated data contained the following fields: age, sex, ethnicity underlying chronic conditions, BMI, and ADL self-performance hierarchy scores. Each de-identified individual-level observation had a unique identifier created specifically for this dataset. The unique identifier enabled the linkage of all data to be aggregated while protecting older adults’ identities. The study population characteristics were expressed as the number and proportion of participants for categorical variables and means with standard deviation (SD) for continuous variables. The prevalence of individual conditions and multi-morbidity were reported as a percentage of participants affected. Prevalence ratios (PR) and 95% confidence intervals (CI) were calculated using Poisson regression with robust variance [32] to assess the associations between BMI and disability in ADL and multi-morbidity. Two adjusted models were constructed: Model 1 adjusted for age and gender, and Model 2 combined model 1 with ethnicity (European, Māori, Asian, Pacific, and other) and District Health Board (DHB) region (Central, Midland, Northern, South Island). The associations between BMI and disability in ADL and multi-morbidity were further stratified by age groups (60-69 years, 70-79 years, 80-89 years and ≥ 90 years). For a sensitivity analysis, a linear regression model was fitted to assess the association between BMI and ADL using scores of ADL (0 = independent to 6 = total dependent).

## Results

### Background characteristics and body mass index

Of the 224,200 resident records obtained, 3859 records were excluded because residents were under 60 years, age was unknown, or had missing information on BMI, ethnicity, DHB region or ADL hierarchy. Thus, the final sample size was 198,790 resident records. Table 1 presents the results of the background characteristics and BMI of the residents. 74.4% were 80 years and over, female (66.1%), and European (90.5%). Among the records studied, less than half (*n* = 83,285, 41.9%) had normal weight. Of these 198,790 resident’s records, 10.6% were underweight, 29.4% were overweight, 16.3% were obese, and 1.7% were extremely obese.

**Table 1:** Background characteristic by body mass index among older adults in nursing homes

Variables	Total		Proportion of BMI					X^2^; df	***P***-value^*ǂ*^
	Frequency (***N*** = 198,790)	Total %	Underweight (***n*** = 21,109) 10.6%	Normal weight (***n*** = 83,285) 41.9%	Overweight (***n*** = 58,543***)*** 29.4%	Obese (***n*** = 32,490) 16.3%	Extremely obese (***n*** = 3363) 1.7%		
**Age, %**									
60–69	11,840	6.0	7.1	27.8	29.0	29.3	6.8	12,054.69; 12	< 0.001
70–79	38,949	19.6	7.1	33.8	31.5	24.0	3.6		
80–89	89,321	44.9	9.9	42.0	30.9	16.1	1.1		
≥ 90	58,680	29.5	14.7	49.9	26.1	9.1	0.3		
**Gender, %**									
Men	67,477	33.9	5.5	40.8	34.6	17.6	1.5	3606.98; 4	< 0.001
Women	131,313	66.1	13.3	42.2	26.8	15.7	1.8		
**Ethnicity, %**									
European	179,874	90.5	10.8	42.4	29.4	15.9	1.5	3407.58; 16	< 0.001
Māori	8459	4.3	4.7	29.0	31.6	29.4	5.3		
Asian	5039	2.5	18.1	50.0	24.9	6.6	0.3		
Pacific	3369	1.7	5.3	34.0	32.2	24.5	4.0		
Other	2049	1.0	8.1	43.6	31.9	15.0	1.5		
**DHB region, %**									
Central	39,111	19.7	11.0	41.1	29.5	16.5	1.9	547.37; 12	< 0.001
Midland	40,665	20.5	10.4	40.3	29.9	17.6	1.8		
Northern	59,208	29.8	11.8	44.0	28.3	14.3	1.6		
South Island	59,806	30.1	9.4	41.4	30.3	17.4	1.6		

*BMI* Body mass index

^ǂ^*P*-values based on Pearson Chi-square test

A significant relationship was found between age and BMI (X^2^ = 12,054.69, df = 12, *p* = < 0.001); the proportion of underweight and normal weight increased with increasing age, and conversely, the proportion of overweight, obese, and extremely obese reduced with increasing age. For example, obese and extremely obese decreased from 36.1% in the 60–69 years to 9.4% in the 90 and over age groups. A meaningful association was found between gender and BMI (X^2^ = 3606.98, df = 4, *p* = < 0.001), the proportion of underweight females was 2.5 times greater than that of males, but men were more highly represented in the overweight category. The gender proportions were similar for normal weight, obese and extremely obese BMI groups. Ethnic groups had different patterns with respect to BMI. The proportion of underweight and normal weight was significantly higher (68.1%) among those who identified as Asian. Proportions of obese and extremely obese were higher (34.7%) for Māori ethnicities than all other ethnic groups, whose proportions ranged from 6.9% for Asians to 28.5% for Pacific people. Although statistically different, the proportions by region indicate a similar BMI mix.

### Disability in activities of daily living among older adults in nursing homes

The prevalence and distribution of ADL hierarchy by demographic characteristics and BMI is presented in Table 2. The prevalence of disability showed that about a quarter of residents were independent, whereas 16.2 and 17.7% required supervision and limited assistance in performing ADL, respectively. Maximal assistance was needed by 8.1%, whereas 16.3% were dependent and 5.2% were totally dependent.

**Table 2:** Hierarchy of activities of daily living among older adults in nursing homes

Variables	Independent^**a**^ (***n*** = 48,738) 24.5%	Hierarchy of ADL						X^**2**^; df	***P***-value ǂ
		Supervision required^**b**^ (***n*** = 32,162) 16.2%	Limited assistance^**c**^ (***n*** = 35,203) 17.7%	Extensive assistance^**d**^(***n*** = 23,716) 11.9%	Maximal assistance^**e**^ (***n*** = 16,072) 8.1%	Dependence^**f**^ (***n*** = 32,463) 16.3%	Total dependence^**g**^ (***n*** = 10,436) 5.2%		
**Age, %**									
60–69	26.9	16.1	15.5	14.7	6.3	13.3	6.6	811.41; 18	< 0.001
70–79	24.3	16.2	16.7	13.1	7.5	15.9	6.4		
80–89	24.5	16.9	17.7	11.6	8.1	16.0	5.2		
≥ 90	24.2	15.1	18.8	11.0	8.8	17.8	4.4		
**Gender, %**									
Men	23.2	16.3	18.4	14.3	8.4	15.3	4.2	859.52; 6	< 0.001
Women	25.2	16.1	17.3	10.7	7.9	16.9	5.8		
**Ethnicity, %**									
European	24.4	16.3	18.0	12.0	8.2	16.2	5.0	706.06; 24	< 0.001
Māori	26.7	16.8	15.2	11.9	6.8	16.0	6.6		
Asian	27.2	14.6	13.6	9.4	8.1	18.8	8.3		
Pacific	19.9	13.4	16.8	11.3	6.9	20.5	11.1		
Other	25.3	12.5	17.7	11.4	7.1	18.1	7.9		
**BMI, %**									
Underweight	16.1	12.7	16.4	10.6	10.3	22.5	11.5	5384.70; 24	< 0.001
Normal	23.0	16.0	17.7	11.7	8.7	16.6	6.3		
Overweight	27.1	17.4	18.0	11.9	7.3	14.7	3.7		
Obese	29.0	16.9	18.0	13.2	6.7	14.4	1.8		
Extremely obese	28.3	13.4	18.0	16.0	7.0	16.2	0.6		

*BMI* Body mass index

ǂ Value based on Pearson chi-square

^a^Independent in all four ADL

^b^Supervision in one ADL

^c^Limited assistance in one or more ADLs

^d^Extensive assistance in personal hygiene or toileting

^e^Extensive assistance in eating or locomotion

^f^Total dependence in eating and/or locomotion

^g^Total dependence in all four ADL

Age was significantly associated with disability in ADL (X^2^ = 811.41, df = 18, *p* = < 0.001); the proportion who were independent decreased with increasing age, whereas the proportion who were totally dependent decreased across age groups. Total dependence was higher among women (5.8%) as compared to men (4.2%). Total dependence in ADL was highest among the Pacific ethnicity (11.1%) and lower among the Māori (6.6%) and European ethnicities (5.0%). Total dependence in ADL decreased across the categories of BMI; 11.5, 1.8 and 0.6% among the underweight, obese, and extremely obese, respectively. On the other hand, the proportion of adults independent in ADL was highest among obese (29.0%) followed by the extremely obese (28.3%) and lowest among the underweight (16.1%).

### Multi-morbidity among older adults in nursing homes

The proportion of older adults with 2 or more chronic conditions was highest (69.5%) among adults 70-79 years and lowest (63.7%) among adults 60-69 years (Table 3). The 5 most common chronic diseases were heart disease (36.4%), dementia (35.6%), depression (24.3%), stroke (23.2%), and diabetes mellitus (19.3%). However, the most common primary diagnosis at the time of admission was dementia (25.9%). Men were more likely (68.9%) to have two or more disease conditions compared to women (61.6%), (X^2^ = 1183.29, df = 2, *p* = < 0.001). A significant relationship was found between ethnicity and the number of disease conditions among older adults (X^2^ = 287.89, df = 8, *p* = < 0.001), with multi-morbidity being highest (70.5%) for Māori ethnicity and lowest (64.1%) for Asian ethnicity. An association was found between increasing BMI and the number of disease conditions among residents (X^2^ = 979.25, df = 8, *p* = < 0.001). The number of older adults with two or more conditions was 59.6, 65.5 and 70.7% among the underweight, overweight, and extremely obese, respectively.

**Table 3:** Number of chronic disease conditions among older adults in nursing homes

Variables	Number of chronic conditions			X^**2**^; df	***P***-value¥
	None (***n*** = 13,130) 7.4%	One (***n*** = 52,432) 28.5%	Two or more^**a**^ (***n*** = 133,228) 64.1%		
**Age, %**					
60–69	6.5	29.8	63.7	3511.11; 6	< 0.001
70–79	4.6	25.9	69.5		
80–89	5.9	27.1	67.1		
≥ 90	11.6	32.3	56.0		
**Gender, %**					
Men	5.5	25.6	68.9	1183.29; 2	< 0.001
Women	8.3	30.1	61.6		
**Ethnicity, %**					
European	7.5	28.7	63.8	287.89; 8	< 0.001
Māori	4.2	25.3	70.5		
Asian	9.5	29.1	61.4		
Pacific	4.8	28.1	67.0		
Other	6.3	31.6	62.1		
**BMI, %**					
Underweight	9.3	31.1	59.6	979.25; 8	< 0.001
Normal	7.9	30.3	61.8		
Overweight	6.8	27.6	65.5		
Obese	5.7	24.7	69.6		
Extremely obese	6.0	23.4	70.7		

¥*P*-Value based on Pearson chi-square

^a^Multi-morbidity

### Association between BMI and multi-morbidity and disability of ADL

The association between BMI and multi-morbidity (in comparison with zero/one condition) is presented in Table 4. The results show no significant differences in multi-morbidity among the various categories of BMI in the crude and adjusted models. The proportion of multi-morbidity was marginally higher among the extremely obese (PR, 95% CI: 1.02, 1.01–1.03), but the effect was attenuated when the model was adjusted for age, gender, ethnicity, and region. In a stratified analysis, the prevalence ratio for multi-morbidity was 10% lower among the extremely obese compared to the normal weight of adults ≥90 years (PR, 95% CI: 0.90, 0.83–0.98).

**Table 4:** Prevalence ratios (95% CI) for the association between body mass index and multi-morbidity

Body mass index	N	Crude		Model 1		Model 2	
		PR (95% CI)	***p***-value	PR (95% CI)	***p***-value	PR (95% CI)	***p***-value
Underweight	21,109	0.99 (0.98, 0.99)	< 0.001	0.99 (0.99, 1.00)	0.001	0.99 (0.99, 1.00)	0.002
Normal (ref)	83,285	1.00		1.00		1.00	
Overweight	58,543	1.01 (1.01, 1.02)	< 0.001	1.01 (1.00, 1.01)	0.001	1.00 (1.00, 1.00)	0.006
Obese	32,490	1.02 (1.02, 1.03)	< 0.001	1.01 (1.01, 1.02)	< 0.001	1.01 (1.01, 1.01)	< 0.001
Extremely obese	3363	1.02 (1.01, 1.03)	< 0.001	1.00 (1.00, 1.01)	0.395	1.00 (0.99, 1.01)	0.783

Model 1: Adjusted for age and gender; Model 2: Model 1 + ethnicity and DHB region

*PR* Prevalence ratio, *Ref* Reference, *N* Number of respondents

As shown in Table 5, BMI was inversely associated with disability (being dependent) in ADL. As compared to normal weight, overweight (PR, 95%CI: 0.58, 0.55–0.61), obese (PR, 95%CI: 0.29, 0.27–0.31) and extremely obese (PR, 95%CI: 0.10, 0.07–0.15) had lower prevalence ratios for disability in ADL. In contrast, being underweight was associated with higher PR for disability in ADL (PR, 95%CI: 1.83, 1.75–1.91). This association was not altered after controlling for age, gender, ethnicity, and region. In a stratified analysis, an inverse association between BMI and disability in ADL was similarly observed amongst all age groups. In a sensitivity analysis, a linear regression model fitted using scores of ADL shows a 0.58 increase in ADL scores for the underweight compared to adults who were normal weight. On the other hand, overweight, obesity and extreme obesity were inversely associated with ADL scores.

**Table 5:** Prevalence ratios (95% CI) for the association between body mass index and disability in ADL

Body mass index	N	Crude		Model 1		Model 2	
		PR (95% CI)	***p***-value	PR (95% CI)	***p***-value	PR (95% CI)	***p***-value
Underweight	21,109	1.83 (1.75, 1.91)	< 0.001	1.80 (1.72, 1.89)	< 0.001	1.81 (1.73, 1.90)	< 0.001
Normal (ref)	83,285	1.00		1.00		1.00	
Overweight	58,543	0.58 (0.55, 0.61)	< 0.001	0.56 (0.53, 0.58)	< 0.001	0.55 (0.53, 0.58)	< 0.001
Obese	32,490	0.29 (0.27, 0.31)	< 0.001	0.25 (0.23, 0.27)	< 0.001	0.25 (0.23, 0.27)	< 0.001
Extremely obese	3363	0.10 (0.07, 0.15)	< 0.001	0.07 (0.05, 0.11)	< 0.001	0.07 (0.05, 0.11)	< 0.001

Model 1: Adjusted for age and gender; Model 2: Model 1 + ethnicity and DHB region

*P.R.* Prevalence ratio, *Ref* Reference, *N* Number of respondents

## Discussion

We examined the prevalence of obesity and its impact on multi-morbidity and disability in ADL in a sample of older New Zealanders aged ≥60 years living in nursing homes. Consistent with previous studies [3, 7, 33], we found more than 60% of the adults to have multi-morbidity of chronic diseases. Of the studied population, 75.4% were at various stages of disablement. Independence in daily living activities was related to weight. Those who were overweight and obese were more independent than those who were underweight. This finding contributes to the ongoing discussion of the so-called ‘obesity paradox’, indicating, against the expectation, that overweight and obesity seems to decrease the risk of functional decline.

A growing number of studies have addressed the relationship between the weight of older adults and the risk of disability in activities of daily living [23]. These studies have suggested that the relationship between BMI and disability in ADL is linear. In addition, studies conducted in nursing homes have demonstrated that being overweight or obese is associated with better functional status [19, 20] than those considered underweight and normal weight [21]. Based on a nationally representative sample of older adults in nursing homes, our study corroborates this relationship. However, we have used data across the entire spectrum of BMI, from underweight (BMI < 18 kg/m^2^) to severe obesity (BMI ≥40kgm^2^) and demonstrate a non-linear relationship between BMI and disability in ADL, with the highest risk of disability being identified among underweight older adults.

Underweight has been considered one of the most critical components of the frailty index [34]. It may represent a higher prevalence of malnutrition or a decrease in muscle mass, resulting in decreased physical strength [35]. In addition, unintentional weight loss may indicate underlying health problems such as cancer or swallow difficulties [36, 37]. The observed association could be due to reverse causality; disability may cause underweight through eating difficulties and challenges in cooking or obtaining foodstuffs, as reported among community-dwelling older adults [38, 39]. This, however, might not be the case in nursing homes where food, and support to eat it, are provided. Therefore, more attention should be paid to institutionalised older adults’ nutritional status and activity levels when studying the association of BMI and ADL. Guidelines on weight management based on data from community-dwelling older adults and younger adult populations should not routinely be adopted for the underweight older adult in nursing homes as there is a lack of evidence to support the practice.

The findings of our study further suggest that the living setting of older adults may make a difference in the reported relationship between BMI and the functional status of older adults. Studies conducted in community settings have identified higher BMI as a risk for functional decline [11–13, 16, 22, 24]. Our findings are not consistent with the above conclusions. However, they support an emerging trend identified among studies conducted in nursing homes that overweight and obesity have a protective effect [19–21]. The apparent difference could be attributable to the differences in the functional background of older adults in the nursing home and community settings [20]. Nursing home residents are a particular group of older individuals with severe functional limitations. How and why being overweight and obese in residential care settings needs to be better understood.

Lastly, differences in study design and the measurement of ADL may account for the findings. For example, studies conducted in nursing homes have mainly been cross-sectional [19, 21], compared to the longitudinal studies [10–13] among community-dwelling participants. The latter has reported that high BMI is a risk for ADL disability. Increased BMI (obesity), when assessed at any given timepoint, is associated with less disability than normal or lower BMI, but when examined over time, a higher BMI becomes a predictor of significant risk of ADL decline over 4–8 years. Thus, longitudinal designs in nursing homes studies might help predict functional decline in an older adult’s life. In addition, previous studies have exclusively relied on either the Barthel [18, 20, 21] or Katz [12, 19, 40] scales to measure ADL. These instruments were developed several decades ago to assess function by *direct observation* of the performance of patients undergoing rehabilitation for musculoskeletal conditions [41, 42]. However, except for Kaiser et al. [20], all studies measuring functional status as an outcome in older adults in the community and nursing homes relied on self-report. Thus, these studies are limited by responder bias as only those who could comprehend complete instruments participated. Our study used direct observation by a clinician over a 3 day ‘look back’ timeframe utilising a standardised framework for calculating the ADL score [43].

### Implications

The current study’s results imply that the burden of increased disability in nursing homes rests with the underweight older population. Given that dementia is the most common primary diagnosis on admission, particular attention needs to be given to reducing any further weight loss and where possible supporting weight gain. This is important in the planning of care and resources. There is an immediate need for policy-makers and practitioners to be aware of and apply appropriate interventions for underweight nursing home residents. The care challenges associated with older adults with obesity were established [44]. However, little is known about the underweight and their experience**;** suggesting that the healthcare sector may be unprepared to address the care needs associated with declining abilities in ADLs. Further research on the care needs of this population is required.

### Strengths and limitations

This study should be interpreted with consideration of its limitations. We utilised a large national database of adults in nursing homes. The use of fixed BMI cutoff points for all ages might result in “differential misclassification bias” due to the reduction in body fatness with age resulting in reduced sensitivity of BMI cutoff points [45]. This could lead to an inaccurate estimation of the increase in the prevalence of obesity with age and the risk of chronic diseases associated with BMI [45] Similarly, the high prevalence of osteoporosis and kyphosis in older adults, particularly those over 80 years old, could overestimate BMI.

We relied on BMI as a metric for adiposity in the study. However, we acknowledge that the inherent limitations of BMI may confound the true association between obesity and functional decline. For example, BMI does not account for the fat distribution and body composition or distinguishes between lean and fat body mass [46, 47]. Therefore, future studies should also use other metrics such as waist circumference, which may help elicit the role of central or visceral accumulation of fat, contributing to disabilities in nursing home residents.

## Conclusion

Close to half of the New Zealand nursing home residents had moderate to severe obesity. A high percentage lived with multi-morbidity of chronic diseases and at various stages of disablement. Being underweight was associated with a decline in the performance of ADL. In contrast, being overweight and obese had positive effects on ADL, confirming that the obesity paradox plays a vital role in this population. Detection and management of underweight in nursing home residents could prevent a decline in ADL. A study that explores the impact of nutritional status, nutrition intake and body composition among nursing home residents is recommended.

## Data Availability

The data supporting findings of this study are available from interRAI New Zealand but restrictions apply to the availability of these data, which were used under license for the current study, and so are not publicly available. Data are however available from the authors upon reasonable request and with the permission of interRAI New Zealand.

## Abbreviations

ADL: Activity of daily living
BMI: Body mass index
DHB: District Health Board
interRAI: International Resident Assessment Instrument

## References

[1] Ministry of Health (2020). Annual update of key results 2019/20: New Zealand health survey.

[2] An KO, Kim J (2016). Association of sarcopenia and obesity with multimorbidity in Korean adults: a nationwide cross-sectional study. J Am Med Dir Assoc.

[3] Kivimäki M, Kuosma E, Ferrie JE, Luukkonen R, Nyberg ST, Alfredsson L (2017). Overweight, obesity, and risk of cardiometabolic multimorbidity: pooled analysis of individual-level data for 120 813 adults from 16 cohort studies from the USA and Europe. Lancet Public Health.

[4] Pérez CM, Sánchez H, Ortiz AP (2013). Prevalence of overweight and obesity and their cardiometabolic comorbidities in Hispanic adults living in Puerto Rico. J Community Health.

[5] Schienkiewitz A, Mensink GBM, Scheidt-Nave C (2012). Comorbidity of overweight and obesity in a nationally representative sample of German adults aged 18-79 years. BMC Public Health.

[6] Cai S, Rahman M, Intrator O (2013). Obesity and pressure ulcers among nursing home residents. Med Care.

[7] Pes GM, Licheri G, Soro S, Longo NP, Salis R, Tomassini G (2019). Overweight: a protective factor against comorbidity in the elderly. Int J Environ Res Public Health.

[8] Zhang L, Ma L, Sun F, Tang Z, Chan P (2020). A multicenter study of multimorbidity in older adult inpatients in China. J Nutr Health Aging.

[9] Neeland IJ, Das SR, Simon DN, Diercks DB, Alexander KP, Wang TY (2017). The obesity paradox, extreme obesity, and long-term outcomes in older adults with ST-segment elevation myocardial infarction: results from the NCDR. Eur Heart J Qual Care Clin Outcomes.

[10] Al Snih S, Ottenbacher KJ, Markides KS, Kuo Y-F, Eschbach K, Goodwin JS (2007). The effect of obesity on disability vs mortality in older Americans. Arch Intern Med.

[11] Busetto L, Romanato G, Zambon S, Calò E, Zanoni S, Corti MC (2009). The effects of weight changes after middle age on the rate of disability in an elderly population sample. J Am Geriatr Soc.

[12] Okamoto S, Okamura T, Sugiyama D, Hayakawa T, Nakamura Y, Miyagawa N (2018). Overweight or underweight and the risk of decline in activities of daily living in a 22-year cohort study of a Japanese sample. Geriatr Gerontol Int.

[13] Tsai H-J, Chang F-K (2017). Associations between body mass index, mid-arm circumference, calf circumference, and functional ability over time in an elderly Taiwanese population. PLoS One.

[14] Walter S, Kunst A, Mackenbach J, Hofman A, Tiemeier H (2009). Mortality and disability: the effect of overweight and obesity. Int J Obes.

[15] Bahat G, Muratlı S, İlhan B, Tufan A, Tufan F, Aydin Y (2015). Body mass index and functional status in community dwelling older Turkish males. Aging Male Off J Int Soc Study Aging Male.

[16] Bahat G, Tufan A, Aydin Y, Tufan F, Bahat Z, Akpinar TS (2015). The relationship of body mass index and the functional status of community-dwelling female older people admitting to a geriatric outpatient clinic. Aging Clin Exp Res.

[17] Chen H, Guo X (2008). Obesity and functional disability in elderly Americans. J Am Geriatr Soc.

[18] Kiesswetter E, Schrader E, Diekmann R, Sieber CC, Volkert D (2015). Varying associations between body mass index and physical and cognitive function in three samples of older adults living in different settings. J Gerontol A Biol Sci Med Sci.

[19] Bahat G, Tufan F, Saka B, Akin S, Ozkaya H, Yucel N (2012). Which body mass index (BMI) is better in the elderly for functional status?. Arch Gerontol Geriatr.

[20] Kaiser R, Winning K, Uter W, Volkert D, Lesser S, Stehle P (2010). Functionality and mortality in obese nursing home residents: an example of ‘risk factor paradox’?. J Am Med Dir Assoc.

[21] Ozturk GZ, Egici MT, Bukhari MH, Toprak D (2017). Association between body mass index and activities of daily living in homecare patients. Pak J Med Sci.

[22] Lv Y-B, Yuan J-Q, Mao C, Gao X, Yin Z-X, Kraus VB (2018). Association of body mass index with disability in activities of daily living among Chinese adults 80 years of age or older. JAMA Netw Open.

[23] Rejeski WJ, Marsh AP, Chmelo E, Rejeski JJ (2010). Obesity, intentional weight loss and physical disability in older adults. Obes Rev.

[24] Cheng FW, Gao X, Bao L, Mitchell DC, Wood C, Sliwinski MJ (2017). Obesity as a risk factor for developing functional limitation among older adults: a conditional inference tree analysis. Obes Silver Spring Md.

[25] Meehan B, McCreadie M. Agreements for use of the InterRAI assessment system. Wellington: InterRAI New Zealand Governance Board; 2016.

[26] InterRAI. interRAI Long-Term Care Facilities (LTCF) assessment form and user’s manual (standard English edition), 10.0. InterRAI; 2020. Available from: https://catalog.interrai.org/LTCF-long-term-care-facilities-manual-standard-english-10.0. Cited 2021 Sep 16.

[27] interRAI assessments - interRAI. 2021. Available from: https://www.interrai.co.nz/about/interrai-assessments-in-new-zealand/. Cited 2021 Jul 21.

[28] InterRAI New Zealand. InterRAI assessment protocols. Wellington: InterRAI; 2019.

[29] Kim H, Jung Y-I, Sung M, Lee J-Y, Yoon J-Y, Yoon J-L (2015). Reliability of the interRAI Long Term Care Facilities (LTCF) and interRAI Home Care (HC). Geriatr Gerontol Int.

[30] Morris JN, Berg K, Fries BE, Steel K, Howard EP. Scaling functional status within the interRAI suite of assessment instruments. BMC Geriatr. 2013;13(1) Available from: https://link.gale.com/apps/doc/A534608702/AONE?sid=bookmark-AONE&xid=df598bb7. Cited 2021 Jul 23. 10.1186/1471-2318-13-128 PMC3840685 24261417

[31] World Health Organization (2011). Waist circumference and waist-hip ratio: report of a WHO expert consultation.

[32] Barros AJ, Hirakata VN (2003). Alternatives for logistic regression in cross-sectional studies: an empirical comparison of models that directly estimate the prevalence ratio. BMC Med Res Methodol.

[33] Ofori-Asenso R, Chin KL, Curtis AJ, Zomer E, Zoungas S, Liew D (2019). Recent patterns of multimorbidity among older adults in high-income countries. Popul Health Manag.

[34] Ensrud KE, Ewing SK, Taylor BC, Fink HA, Cawthon PM, Stone KL (2008). Comparison of 2 frailty indexes for prediction of falls, disability, fractures, and death in older women. Arch Intern Med.

[35] Fisher AL (2004). Of worms and women: sarcopenia and its role in disability and mortality. J Am Geriatr Soc.

[36] Chau D, Cho LM, Jani P, St Jeor ST (2008). Individualizing recommendations for weight management in the elderly. Curr Opin Clin Nutr Metab Care.

[37] Kane RL, Shamliyan T, Talley K, Pacala J (2012). The association between geriatric syndromes and survival. J Am Geriatr Soc.

[38] Janssen I, Heymsfield SB, Ross R (2002). Low relative skeletal muscle mass (sarcopenia) in older persons is associated with functional impairment and physical disability. J Am Geriatr Soc.

[39] Portegijs E, Rantakokko M, Viljanen A, Sipilä S, Rantanen T (2016). Identification of older people at risk of ADL disability using the life-space assessment: a longitudinal cohort study. J Am Med Dir Assoc.

[40] Larrieu S, Pérès K, Letenneur L, Berr C, Dartigues JF, Ritchie K (2004). Relationship between body mass index and different domains of disability in older persons: the 3C study. Int J Obes Relat Metab Disord J Int Assoc Study Obes.

[41] Katz S, Ford AB, Moskowitz RW, Jackson BA, Jaffe MW (1963). Studies of illness in the aged: the index of ADL: a standardized measure of biological and psychosocial function. JAMA.

[42] Mahoney FI, Barthel DW (1965). Functional evaluation: the Barthel index: a simple index of independence useful in scoring improvement in the rehabilitation of the chronically ill. Md State Med J.

[43] InterRAI New Zealand (2020). Assessor workbook. InterRAI assessments. Education and support services.

[44] Bradway C, DiResta J, Miller E, Edmiston M, Fleshner I, Polomano R (2009). Caring for obese individuals in the long-term care setting | population health learning network. An Long-Term Care.

[45] Baumgartner RN, Heymsfield SB, Roche AF (1995). Human body composition and the epidemiology of chronic disease. Obes Res.

[46] Antonopoulos AS, Oikonomou EK, Antoniades C, Tousoulis D (2016). From the BMI paradox to the obesity paradox: the obesity-mortality association in coronary heart disease. Obes Rev Off J Int Assoc Study Obes.

[47] Villareal DT, Apovian CM, Kushner RF, Klein S, American Society for Nutrition, NAASO, The Obesity Society (2005). Obesity in older adults: technical review and position statement of the American Society for Nutrition and NAASO, The Obesity Society. Am J Clin Nutr.

